# Oleaginous yeast *Yarrowia lipolytica* culture with synthetic and food waste-derived volatile fatty acids for lipid production

**DOI:** 10.1186/s13068-017-0942-6

**Published:** 2017-10-30

**Authors:** Ruiling Gao, Zifu Li, Xiaoqin Zhou, Shikun Cheng, Lei Zheng

**Affiliations:** School of Energy and Environmental Engineering, Beijing Key Laboratory of Resource-oriented Treatment of Industrial Pollutants, University of Science and Technology Beijing, Beijing, 100083 People’s Republic of China

**Keywords:** Microbial lipids, *Yarrowia lipolytica*, Volatile fatty acids, Food waste fermentation, Biodiesel

## Abstract

**Background:**

The sustainability of microbial lipids production from traditional carbon sources, such as glucose or glycerol, is problematic given the high price of raw materials. Considerable efforts have been directed to minimize the cost and find new alternative carbon sources. Volatile fatty acids (VFAs) are especially attractive raw materials, because they can be produced from a variety of organic wastes fermentation. Therefore, the use of volatile fatty acids as carbon sources seems to be a feasible strategy for cost-effective microbial lipid production.

**Results:**

Lipid accumulation in *Y. lipolytica* using synthetic and food waste-derived VFAs as substrates was systematically compared and evaluated in batch cultures. The highest lipid content obtained with acetic, butyric, and propionic acids reached 31.62 ± 0.91, 28.36 ± 0.74, and 28.91 ± 0.66%, respectively. High concentrations of VFA inhibited cell growth in the following order: butyric acid > propionic acid > acetic acid. Within a 30-day experimental period, *Y. lipolytica* could adapt up to 20 g/L acetic acid, whereas the corresponding concentration of propionic acid and butyric acid were 10 and 5 g/L, respectively. Cultures on a VFA mixture showed that the utilization of different types of VFA by *Y. lipolytica* was not synchronized but rather performed in a step-wise manner. Although yeast fermentation is an exothermic process, and the addition of VFA will directly affect the pH of the system by increasing environmental acidity, cultures at a cultivation temperature of 38 °C and uncontrolled pH demonstrated that *Y. lipolytica* had high tolerance in the high temperature and acidic environment when a low concentration (2.5 g/L) of either synthetic or food waste-derived VFA was used. However, batch cultures fed with food fermentate yielded lower lipid content (18.23 ± 1.12%) and lipid productivity (0.12 ± 0.02 g/L/day). The lipid composition obtained with synthetic and food waste-derived VFA was similar to commercial biodiesel feedstock.

**Conclusions:**

This work demonstrated the feasibility of utilizing synthetic and food waste-derived VFA for lipid production by *Y. lipolytica*. The good adaptability of *Y. lipolytica* to the high temperature and acidic environment further illustrated its considerable potential for practical application.

## Background

As a means to address the global energy crisis and climate change, biodiesel has received increasing attention from industry, academia, and governments because of its potential as a renewable energy resource that could replace conventional fossil fuels [1]. Biodiesel, a mixture of fatty acid alkyl esters, could be produced via transesterification from numerous oil sources, such as vegetable and animal oils or fats and cooking oil waste [2, 3]. The sustainability of biodiesel production from these sources is problematic given its production limitations and expensive raw materials [4]. In particular, the use of edible oil for biodiesel production competes with the food industry [5]. A major issue in biodiesel commercialization is finding an inexpensive and sufficient alternate feedstock to improve the long-term sustainability of biodiesel production [6]. Among the potential lipid sources of biodiesel, microbial lipid, which is also known as single-cell oil and produced by oleaginous microorganisms, is the most promising [7, 8].

To date, most studies on lipid production by oleaginous microorganisms have been carried out with glucose as a carbon source. The bioconversion of simple sugars into microbial lipids by oleaginous yeasts is a well-known process [9, 10]. The most competitive yeasts, such as *Yarrowia lipolytica, Lipomyces starkeyi, Cryptococcus curvatus,* and *Rhodosporidium toruloides,* accumulate 40–70% lipids of their dry mass with glucose or glycerol as a carbon source under particular culture conditions, e.g., nitrogen [11], phosphate [12], or sulfate [13] limitations. Despite its efficient transformation into lipids, glucose is an expensive substrate ($500 per ton) and accounts for 60% of the total production cost and 80% of the total medium cost [1]. Therefore, considerable efforts have been directed to minimize the cost of carbon sources and to find new alternative carbon sources. Volatile fatty acids (VFAs) are especially attractive raw materials because they can be produced from the fermentation of a variety of organic wastes [14]. Fei et al. [1] calculated that 1 ton of VFAs produced from food wastes costs USD 30, which is less than 10% of the cost of one ton of glucose. Microbial lipid production from VFAs seems to be a feasible strategy for cost-effective biodiesel production and could also contribute to waste utilization and remediation.

In recent years, taking volatile fatty acids (VFA) as carbon sources for lipid production is becoming a research hotspot [15–17]. As shown in Table 1, under suitable culture conditions, a 0.3–1.3 g/L lipid concentration and a 10–40% lipid content could be obtained by the above-mentioned yeasts in batch culture, and results considerably improved in fed-batch culture. Previous studies focused on the investigation of the optimal culture condition and the corresponding lipids production by using low concentrations of VFA mixtures or acetate as carbon source. However, the lipid production capacity of different types of VFA has not been systematically investigated and evaluated. The factors that influence the VFA output from fermentation, such as temperature [18], pH [19], reactor design [20, 21], are well understood, and increasing the targeted VFA production via process manipulations is possible [17]. Therefore, investigating the effect of one of the specific acids on cell growth and lipid accumulation is necessary. In addition, the inhibitory effect of high VFA concentrations on cell growth, which was considered an important challenge to obtain high yield, was reported in previous studies [1, 14, 16]. According to Fontanille et al. [16], this inhibition was observed for an initial concentration higher than 5 g/L, with VFA mixtures (acetic, propionic, and butyric acids) as carbon source. Whether different VFA types and initial concentrations could also exert inhibitory effects that differ in intensity is of great interest.

**Table 1:** Lipid production from volatile fatty acids by various microorganisms

Strain	Culture mode	Carbon source (g/L)	Culture duration (h)	Lipid conc.(g/L)	Lipid content (wt %)	References
*C. curvatus*	Batch	Mannitol (4.70 g/L) + VFAs (9.30 g/L)	84 h	1.30	48.30	[45]
*C. curvatus*	Batch	Acetate (33 g/L)	60 h	1.04	37.2	[46]
*C. curvatus*	Batch	Acetate (5 g/L)	96 h	0.95	47.3	[15]
	Fed-batch	Glucose (15 g/L) + acetate (5 g/L)	96 h	2.4	50.89	
*C. curvatus*	Fed-batch	VFAs (9.27 g/L)	3, 5, 7 days	1.36	61.00	[5]
*C. curvatus*	Fed-batch	Acetate (5 g/L)	96 h	28.4	60	[10]
*C. curvatus*	Fed-batch	Acetate (30 g/L)	60 h	3.35	66.40	[34]
*C. curvatus*	Fed-batch	Acetate (700 g/L)	72 h	37	53.8	[35]
*C. albidus*	Batch	VFAs (6.5 g COD/L)	96 h	0.31	28.3	[17]
*C. albidus*	Batch	VFAs (2 g/L)	96 h	0.334	27.8	[1]
*C. albidus*	Batch	Acetate (6 g/L)	96 h	0.74	25.8	[47]
	Batch	VFAs (6 g/L)	96 h	0.65	25.1	
*Y. lipolytica*	Batch	Acetate (4 g/L)	96 h	0.56	13.30	[29]
	Batch	Propionate (4 g/L)	96 h	0.31	8.90	
*Y. lipolytica*	Fed-batch	Acetate (12 g/L)	150 h	1.84	30.76	[16]
	Fed-batch	Glucose (24.36 g/L) + acetate (46.53 g/L)	22 h + 46 h	7.35	40.70	
*Y. lipolytica*	Batch	Synthetic VFAs (2.5 g/L)	72 h	0.68	31.69	This study
	Batch	FW-derived VFAs (2.5 g/L)	72 h	0.37	18.23	

Microorganisms possess a series of adaptive mechanisms to respond and adapt to harsh environments. Yeast strains with better adaptive faculty to specific and practical conditions are more conducive for industrial applications. To further explore the feasibility and applicability of yeast-mediated lipid production from VFAs, studies on the tolerance of oleaginous yeast for a high temperature or acidic environments could have considerable significance. Yeast fermentation process is an exothermic process. Banat et al. [22] reported that the system temperature could increase to approximately 11 °C because of the heat released by microbial growth and metabolism in the absence of a cooling system. However, the optimal temperature range of traditional yeast fermentation is 25–30 °C and usually does not exceed 35 °C. The growth of yeast will be suppressed at ambient temperatures over 35 °C [23]. Thus, normal production in high temperature regions and seasons is difficult to guarantee. Research and application of high temperature-resistant yeast in the fermentation industry have received increasing attention worldwide. In addition to temperature, pH is an important factor that significantly influences the cell growth and lipid accumulation of oleaginous microorganisms [8]. The amount of VFAs used as carbon source will directly affect the pH of the system by increasing environmental acidity and decreasing the pH level. Strains with stronger acid tolerance can withstand a larger impact load of VFA concentration to ensure normal biomass and lipids production. Furthermore, using strains with strong tolerance for acidic pH could reduce the requirements of system pH regulation, thereby saving expenses on alkali. Thus, studying the tolerance of strains for acidic environments is crucial to achieving high productivity.

*Yarrowia lipolytica* is considered a model organism for biolipid production because of its ability to accumulate high levels of lipids and its suite of efficient genetic tools [24]. This yeast has high applied potential, both in the production of typical biofuel lipids [25] and oils with unusual fatty acid profiles or polyunsaturated fatty acids [26–28]. The principal objective of this study was to investigate the bioconversion of VFA to lipids by the oleaginous yeast *Y. lipolytica* and the tolerance of the yeast to high temperature and a specific acidic environment. Comparative cultures on food waste fermentate were carried out to evaluate the potential for practical application.

## Methods

### Strain and inoculum preparation

*Yarrowia lipolytica* (CICC 31596) was obtained from the China Center of Industrial Culture Collection (People’s Republic of China) for type culture. The strain was maintained at 4 °C on YPD agar slant (2 mass% agar powder) and subcultured monthly. YPD medium, which is used for the preservation and pre-culture of the seed cells, is composed of 20 g/L glucose, 20 g/L peptone, and 10/L g yeast extract.

For pre-culture, yeast strains were inoculated into YPD medium under 28 °C at 180 rpm for 24 h. The resulting cultures were re-inoculated in YPD medium at a ratio of 10% (v/v) and cultivated under the same conditions for another 24 h. After reaching logarithmic growth phase, *Y. lipolytica* seed culture medium was inoculated at a ratio of 10% (v/v) into the medium for flask batch culture experiments. All the equipment and solutions for microbial cultivation were steam-sterilized by autoclaving at 121 °C for 20 min.

### Batch cultures on synthetic VFA

Batch cultures were performed in 250-mL Erlenmeyer flasks that contained a 100 mL medium. The nutrient medium for all the experiments on synthetic VFA contained, per liter: 1 g NH_4_Cl, 3 g KH_2_PO_4_, 1 g MgSO_4_7H_2_O, 15 mg FeCl_3_·6H_2_O, 7.5 mg ZnSO_4_·7H_2_O, and 0.5 mg CuSO_4_·5H_2_O. Synthetic volatile fatty acids were used as the sole carbon and energy sources in addition to the nutrient medium. The pH of the medium was adjusted using 2 mol/L HCl and NaOH solutions when needed. All cultures were incubated on a rotary shaker at 180 rpm.

To investigate the influence of VFAs on cell growth and lipid accumulation of *Y. lipolytica*, the types and initial concentration of VFAs in the cultures were compared at a cultivation temperature of 28 °C and an initial pH of 6.0. The abovementioned temperature and pH were chosen based on previous studies [16, 29] and were considered the optimum culture conditions. As typical VFA products of anaerobic acidification, acetic, butyric, and propionic acids were tested at initial concentrations of 2.5, 5, 10, and 20 g/L, respectively.

To evaluate the tolerance of *Y. lipolytica* to high temperature and specific acidic environment, comparative studies were carried out on batch cultures at a cultivation temperature of 38 °C and on batch cultures with no pH adjustment.

To identify the utilization of each acid by *Y. lipolytica* in a VFA mixture system, batch culture on a synthetic 5:2:3 VFA mixture of acetic: butyric: propionic acid was conducted at an initial VFA concentration of 2.5 g/L under optimum culture conditions (28 °C, initial pH 6.0). The abovementioned ratio was chosen based on the ratio of these three types of volatile fatty acid in food waste-derived VFA (Table 4) in this study.

### Anaerobic fermentation of food waste (FW)

Anaerobic fermentation of food waste (FW) was performed in an anaerobic digestion (AD) reactor with working volume of 1.5 L. The reactor was incubated at 37 ± 1 °C with minimum rotation. To prepare the feed, food waste was derived from the Dongcun solid waste treatment plant (Beijing), which collects food waste from restaurants in the city. The food waste was blended in a mixer and suitably diluted using de-ionized (DI) water to 6% TS. Mesophilic anaerobic sludge, obtained from an AD reactor in a FW treatment plant in Beijing, China, was used as inoculum after sedimentation for 3 days. An inoculum-to-substrate ratio (ISR) of 0.5, which was the volatile solids (VS) ratio, was used to produce excess organic acids for hydrolysis and acidification and to inhibit successive methanogenesis. The initial pH was adjusted to 7, but it was not controlled during the process. After 5 days of fermentation, when hydrolysis and acidogenesis were finished, the effluent was collected and centrifuged at 4000 rpm for 10 min to remove the suspended solids, and the liquid supernatant was filtrated through a 0.45-μm membrane to be used as the carbon source for *Y. lipolytica* cultivation.

### *Y. lipolytica* culture with FW-derived VFA

The supernatant of FW fermentation effluent, without any additional nutrients, was used as the feedstock for lipid production by *Y. lipolytica*. The culture was conducted in 250-mL Erlenmeyer flasks that contained a 100 mL medium, at a cultivation temperature of 28 °C and an initial pH 6.0. The supernatant was sterilized and suitably diluted using de-ionized (DI) water to achieve a final concentration of 2.5 g VFA/L, to prevent the inhibition of high VFA concentrations, and to be compared with cultures on 2.5 g/L synthetic VFA mixture.

Comparative batch cultures at a cultivation temperature of 38 °C and batch cultures without any pH adjustment were also used to evaluate the lipid production and the tolerance of *Y. lipolytica* to high temperature and specific acidic environment when FW-derived VFA was the carbon source.

### Experimental period

Microorganisms possess a series of adaptive mechanisms to respond and adapt to harsh environments. Adaptation to environmental change requires a lag phase, which is a process characterized by a relatively quiescent period of cell growth. During this lag phase, microbial cells undergo various acclimation regulations in various aspects. Once fully adapted, the cells begin to undergo division. Based on previous researchers’ experimental results for oleaginous yeast, as concluded in Table 1, the culture duration for all batch cultures was standardized to 3 or 4 days after inoculation. However, during such a short period, the growth data of cultures under unfavorable cultivation conditions could be incomplete because of the relatively longer acclimation process (lag phase) of the yeast to restore growth and development.

To obtain a more comprehensive and in-depth evaluation of the biomass production, lipid production, and adaptability of *Y. lipolytica* under unfavorable but practical cultivation conditions, we extended the experimental observation period to 30 days. All cultures were continuously cultivated within the 30-day experimental period until a measurable biomass production was obtained. The cells were harvested at the end of the stationary phase, which was identified by analyzing the OD600 value of culture broth. All culture experiments were performed in duplicate or triplicate.

### Analytical methods

For food waste fermentation, TS, VS, and pH were measured according to the standard method [30]. Suspended solids were separated from the fermentation effluent via centrifugation at 5000 rpm for 10 min, the liquid supernatant was filtrated through a 0.45-μm membrane, soluble COD (SCOD), total nitrogen (TN), and VFAs were measured. The SCOD was analyzed following the HACH method. VFAs were determined using GC (Shimadzu, GC-2014) with a flame ionization detector and capillary column (Stabilwax-DA, 30 m × 0.25 mm × 0.25 μm). The temperature program followed that of previous methods [31].

For *Y. lipolytica* cultivation, a sterile pipette was used to periodically withdraw 1.5-mL samples from the batch cultures. To describe cell growth, cell concentrations were measured as the absorbance of the culture broth at 600 nm (OD600, DR-6000 spectrophotometer, HACH). Biomass production was expressed as DCW (dry cell weight). Cells were harvested from 10 mL of culture medium by centrifugation at 10,000 rpm for 10 min; washed with ethanol (95%) and hexanes to remove extracellular fatty acids [32]; and dried to constant weight at 105 °C in an oven, and then weighed.

Lipids were extracted in accordance with the method of Bligh and Dyer [32] with modifications [33]. Lipids were extracted from lyophilized biomass using a mixture of chloroform and methanol (2:1 v/v). The lipid mixture was centrifuged at 5000*g* for 25 min to completely dissolve the lipids in the solvent. Then, the organic phase was washed twice with 0.15% (w/v) NaCl solution. The purified chloroform layer was evaporated to dryness with nitrogen gas (Nitrogen Evaporation System). Lipid production was expressed as gram lipid per liter culture media. The lipid content was expressed as gram lipid per gram DCW (%).

The fatty acid composition of lipids was determined by GC analysis of FAMEs. FAMEs were converted from fatty acids via saponification followed by methylation in accordance with the method of [34]. FAMEs were analyzed by using a GC-8600 gas chromatograph (Tianpu Instrument Co., Ltd., Beijing, China) equipped with a flame-ionized detector and CP-Sil 88 capillary column (Agilent, USA, 60 m × 0.25 mm × 0.36 µm). The column temperature was maintained at 190 °C for 10 min, increased from 190 to 240 °C at a rate of 10 °C/min, and maintained at 240 °C for 15 min. Hydrogen was used as the carrier gas at 1.0 mL/min. The split ratio was 1:30 (v/v). The injector and the detector temperatures were set at 270 and 300 °C, respectively. Fatty acids were identified by comparing their retention times with those of standard solutions, then quantified based on their respective peak areas and normalized.

To further confirm the cell growth and lipid accumulation in *Y. lipolytica*, fluorescent testing was carried out with laser scanning fluorescence microscopy (LSCM). An amount of 10 μL cell sample from batch cultures was carefully collected at the end of the stationary phase, subsequently stained with 10 μL Nile Red, and imaged with laser scanning confocal microscopy (Leica TCS SP2) under an exciting wavelength of 543 nm.

## Results and discussion

### Effect of VFA types and initial concentrations on cell growth and lipid production

The effects of different VFA types and initial concentrations on the microbial conversion of volatile fatty acids to lipid by the oleaginous yeast *Y. lipolytica* were evaluated in flask batch cultures under optimal culture conditions (28 °C, initial pH 6.0) based on previous studies [1, 14, 17]. Acetic, butyric, and propionic acids, which are all typical VFA products of anaerobic acidification, were investigated at initial concentrations of 2.5, 5, 10, and 20 g/L, respectively. All the volatile fatty acids selected for this study could be used as carbon sources for the cell growth and lipid accumulation of *Y. lipolytica* (Table 2A). The highest lipid contents of the cultures on acetic, butyric, and propionic acids reached 31.62 ± 0.91, 28.36 ± 0.74, and 28.91 ± 0.66% and the average rates of lipid production over the fermentation period reached 0.26 ± 0.02, 0.20 ± 0.01, and 0.18 ± 0.01 g/(L day), at initial VFA concentrations of 5, 2.5, and 2.5 g/L, respectively. It was also found that biomass and lipid productivities obtained with low concentration (2.5 g/L) of VFAs, especially with acetic acid, were close to those achieved with glucose.

**Table 2:** Biomass and lipid production of *Y. lipolytica* on synthetic VFA under different culture conditions

Carbon source	Biomass (g/L)	Lipid conc. (g/L)	Lipid content (wt %)	*Y*_*X*/*S*_ (g/g)	*Y*_*L*/*S*_ (g/g)	Lag phase (day)
(A) 28 °C, initial pH 6.0, 180 rpm						
Glucose (g/L)						
2.5	2.363 ± 0.055	0.880 ± 0.015	37.27 ± 0.63	0.945 ± 0.022	0.352 ± 0.006	< 3 h
Acetic acid (g/L)						
2.5	2.327 ± 0.074	0.724 ± 0.016	31.12 ± 1.05	0.931 ± 0.030	0.290 ± 0.006	< 3 h
5	3.257 ± 0.124	1.036 ± 0.025	31.62 ± 0.91	0.651 ± 0.024	0.207 ± 0.005	< 3 h
10	4.587 ± 0.162	1.174 ± 0.029	25.80 ± 1.22	0.459 ± 0.016	0.117 ± 0.003	1 ± 0
20	7.443 ± 0.303	0.992 ± 0.045	13.13 ± 1.42	0.372 ± 0.015	0.050 ± 0.002	5 ± 2
Propionic acid (g/L)						
2.5	2.147 ± 0.089	0.610 ± 0.018	28.36 ± 0.74	0.859 ± 0.036	0.244 ± 0.007	< 3 h
5	2.500 ± 0.102	0.675 ± 0.010	27.38 ± 0.46	0.500 ± 0.020	0.135 ± 0.002	1 ± 0
10	3.137 ± 0.220	0.676 ± 0.037	22.08 ± 1.21	0.314 ± 0.022	0.068 ± 0.004	9 ± 1
20	N.D.	N.D.	N.D.	N.D.	N.D.	N.D.
Butyric acid (g/L)						
2.5	1.917 ± 0.065	0.548 ± 0.015	28.91 ± 0.66	0.767 ± 0.026	0.219 ± 0.006	< 3 h
5	2.800 ± 0.132	0.554 ± 0.020	19.53 ± 1.08	0.560 ± 0.026	0.111 ± 0.004	2 ± 0
10	N.D.	N.D.	N.D.	N.D.	N.D.	N.D.
20	N.D.	N.D.	N.D.	N.D.	N.D.	N.D.
(B) 38 °C, initial pH 6.0, 180 rpm						
Glucose (g/L)						
2.5	2.290 ± 0.082	0.758 ± 0.032	33.12 ± 0.85	0.916 ± 0.033	0.303 ± 0.012	< 3 h
Acetic acid (g/L)						
2.5	2.307 ± 0.080	0.702 ± 0.020	30.78 ± 0.85	0.923 ± 0.032	0.281 ± 0.008	1 ± 0
5	2.405 ± 0.124	0.722 ± 0.023	30.12 ± 1.20	0.481 ± 0.025	0.144 ± 0.005	2 ± 0
10	2.780 ± 0.105	0.803 ± 0.035	29.12 ± 0.79	0.278 ± 0.011	0.080 ± 0.004	2 ± 0
20	3.077 ± 0.150	0.566 ± 0.033	18.89 ± 1.51	0.154 ± 0.008	0.028 ± 0.002	9 ± 1
Propionic acid (g/L)						
2.5	1.290 ± 0.056	0.363 ± 0.015	28.03 ± 0.74	0.516 ± 0.022	0.145 ± 0.006	2 ± 0
5	1.400 ± 0.082	0.314 ± 0.021	22.61 ± 1.07	0.280 ± 0.016	0.063 ± 0.004	6 ± 1
10	N.D.	N.D.	N.D.	N.D.	N.D.	N.D.
20	N.D.	N.D.	N.D.	N.D.	N.D.	N.D.
Butyric acid (g/L)						
2.5	1.333 ± 0.070	0.347 ± 0.022	26.07 ± 1.58	0.532 ± 0.028	0.139 ± 0.009	2 ± 0
5	1.430 ± 0.109	0.232 ± 0.038	16.82 ± 1.53	0.286 ± 0.022	0.046 ± 0.008	24 ± 3
10	N.D.	N.D.	N.D.	N.D.	N.D.	N.D.
20	N.D.	N.D.	N.D.	N.D.	N.D.	N.D.
(C) 28 °C, pH uncontrolled, 180 rpm						
Glucose (g/L)						
2.5	2.333 ± 0.060	0.802 ± 0.020	34.46 ± 0.82	0.933 ± 0.024	0.320 ± 0.008	< 3 h
Acetic acid (g/L)						
2.5	1.811 ± 0.070	0.469 ± 0.014	26.12 ± 1.16	0.724 ± 0.028	0.188 ± 0.006	1 ± 0
5	2.862 ± 0.125	0.675 ± 0.021	23.55 ± 1.07	0.572 ± 0.025	0.135 ± 0.004	13 ± 2
10	N.D.	N.D.	N.D.	N.D.	N.D.	N.D.
20	N.D.	N.D.	N.D.	N.D.	N.D.	N.D.
Propionic acid (g/L)						
2.5	1.784 ± 0.093	0.351 ± 0.029	19.56 ± 1.42	0.714 ± 0.037	0.140 ± 0.012	6 ± 1
5	N.D.	N.D.	N.D.	N.D.	N.D.	N.D.
10	N.D.	N.D.	N.D.	N.D.	N.D.	N.D.
20	N.D.	N.D.	N.D.	N.D.	N.D.	N.D.
Butyric acid (g/L)						
2.5	1.823 ± 0.160	0.266 ± 0.040	15.22 ± 1.96	0.729 ± 0.064	0.106 ± 0.016	17 ± 2
5	N.D.	N.D.	N.D.	N.D.	N.D.	N.D.
10	N.D.	N.D.	N.D.	N.D.	N.D.	N.D.
20	N.D.	N.D.	N.D.	N.D.	N.D.	N.D.

All the results presented are the mean values ± SD for three independent replicates

Biomass was harvested at the end of the stationary phase, which was identified by analyzing the OD600 value of culture broth, within an observation period of 30 days. Ammonia chloride with an initial concentration of 260 mg-N/L was used as a nitrogen source for *Y. lipolytica*. Except for the cell growth N.D. cultures, all cultures completely consumed carbon sources and nitrogen sources during the harvesting of biomass

*N.D.* not detected; *Y*_*X/S*_ growth yield coefficient, g DCW/g VFAs; *Y*_*L/S*_ lipid yield coefficient, g lipid/g VFAs; *Lag phase* the period from the inoculation to the start of exponential phase

High initial carbon source concentrations could help achieve high cell densities and lipid accumulation in batch culture [1]. Nevertheless, previous studies have reported that microbial growth is inhibited by an initial VFA concentration that exceeds 5 g/L [16]. According to the results of this study, different VFA types and initial concentrations exerted different inhibitory effects on cell growth and lipid accumulation. The biomass and lipid production of *Y. lipolytica* were qualitatively more resistant to acetic acid than to butyric and propionic acids, especially at high initial VFA concentrations.

**Fig. 1:**
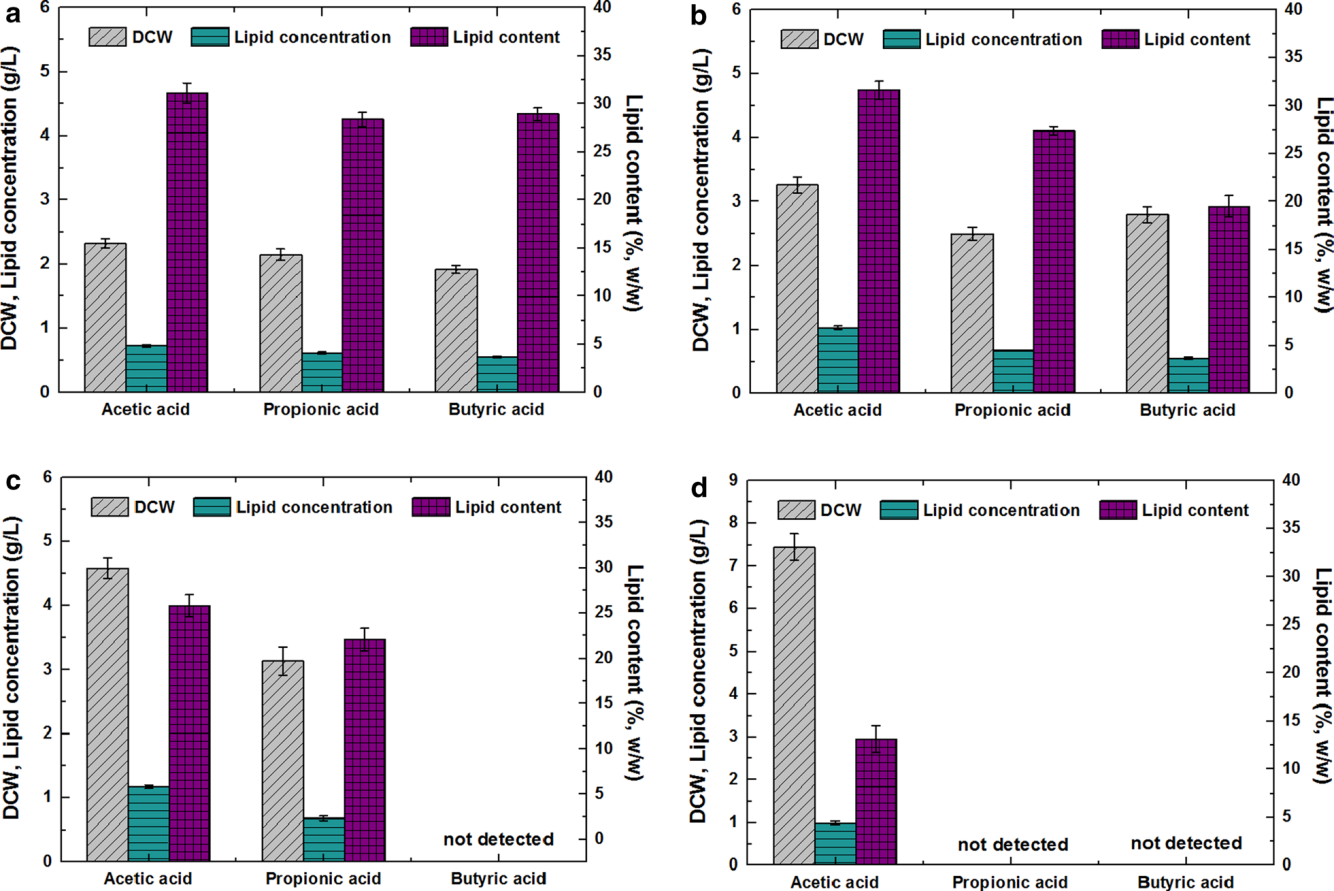
Effects of VFA types and initial concentrations on cell growth and lipid accumulation of *Y. lipolytica* during batch cultivation. The initial VFA concentrations were **a** 2.5 g/L, **b** 5 g/L, **c** 10 g/L, and **d** 20 g/L

In this study, cultures on acetic, butyric, and propionic acids at an initial VFA concentration of 2.5 g/L exhibited highly similar values for biomass production and lipid accumulation (Table 2A, Fig. 1). At this low concentration, *Y. lipolytica* with each type of VFA as carbon sources started cell proliferation without an obvious lag phase. Given that higher initial VFA concentrations exerted stronger inhibitory effects on growth, the yeast would require a longer lag phase prior to effective cell growth and lipid production. Batch cultures on 20 g/L propionic acid, 10 and 20 g/L butyric acid could not achieve biomass and lipid production during the experimental observation period of 30 days. By contrast, cultures adapted to 20 g/L initial acetic acid concentration and began accumulating biomass and lipids after a 5-day adaptive phase. This occurrence indicated that high concentrations of different volatile fatty acids exerted inhibitory effects on *Y. lipolytica* in the following order: butyric acid > propionic acid > acetic acid. *Y. lipolytica* showed a preference for acetic acid for both biomass and lipid production. The same preference is exhibited by *Cryptococcus albidus* and *Candida tropicalis*, which are well known for their abilities to grow on fatty acids [1, 35].

**Fig. 2:**
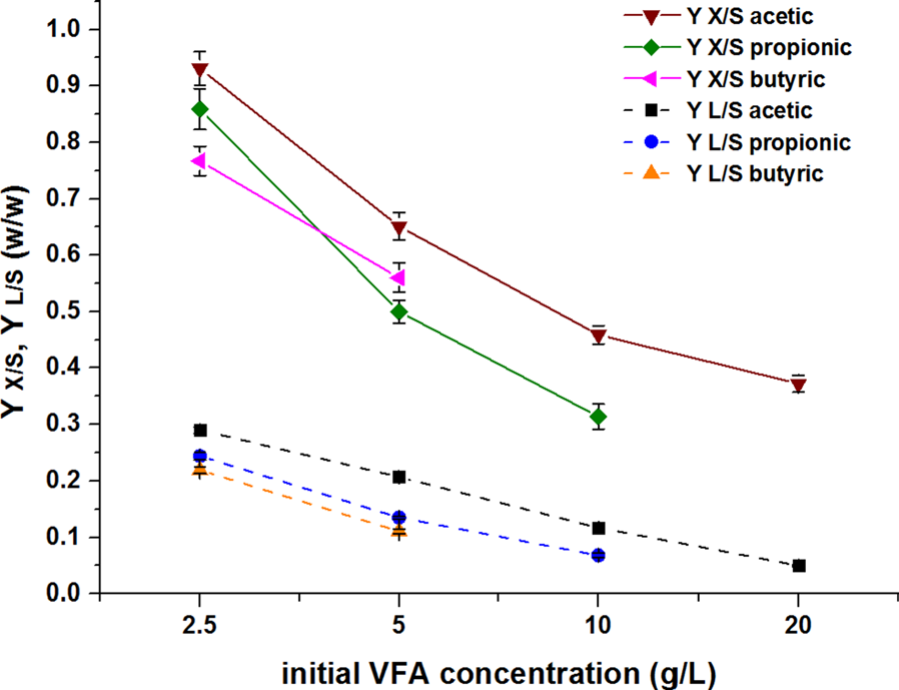
Profile of growth yield coefficient (*Y*_*X*/*S*_) and lipid yield coefficient (*Y*_*L*/*S*_) of *Y. lipolytica* during batch cultivation with different types and concentrations of VFAs

Importantly, VFA concentration also had a significant effect on growth yield coefficient (*Y*_*X*/*S*_) and lipid yield coefficient (*Y*_*L*/*S*_). As shown in Fig. 2, *Y*_*X*/*S*_ and *Y*_*L*/*S*_ decreased asymptotically at higher initial VFA concentrations. The maximum values of both *Y*_*X*/*S*_ and *Y*_*L*/*S*_, which were 0.931 ± 0.030 g DCW/g VFAs and 0.290 ± 0.006 g lipid/g VFAs, respectively, were obtained at an initial acetic acid concentration of 2.5 g/L. In addition, compared with *Y*_*X*/*S*_, *Y*_*L*/*S*_ decreased more rapidly with increasing initial acid concentration. Higher VFA concentrations drastically inhibited *Y*_*L*/*S*_. According to Rodrigues and Pais [35], high initial VFA concentrations inhibit cell growth by chemically interfering with the membrane transport of phosphate, thereby increasing ATP expenditure and consequently inhibiting lipid accumulation. Hence, *Y. lipolytica* cells grew well when a low initial VFA concentration was used as a sole carbon and energy source for lipid production in batch cultures. Although certain ranges of high initial carbon source concentrations could help achieve high cell densities and lipid concentrations, *Y*_*X*/*S*_ and *Y*_*L*/*S*_ should be considered for economical and practical application analysis.

### VFA utilization by *Y. lipolytica* in VFA mixture

An actual anaerobic fermentation stream usually contains all the above types of volatile fatty acids. To determine whether a mutual influence occurs between different types of VFA and how the oleaginous *Y. lipolytica* use them when a mixture of VFA was used as carbon source, a culture on a 2.5 g/L VFA mixture with ratio of 5:2:3 was conducted and compared with cultures on each acid.

**Fig. 3:**
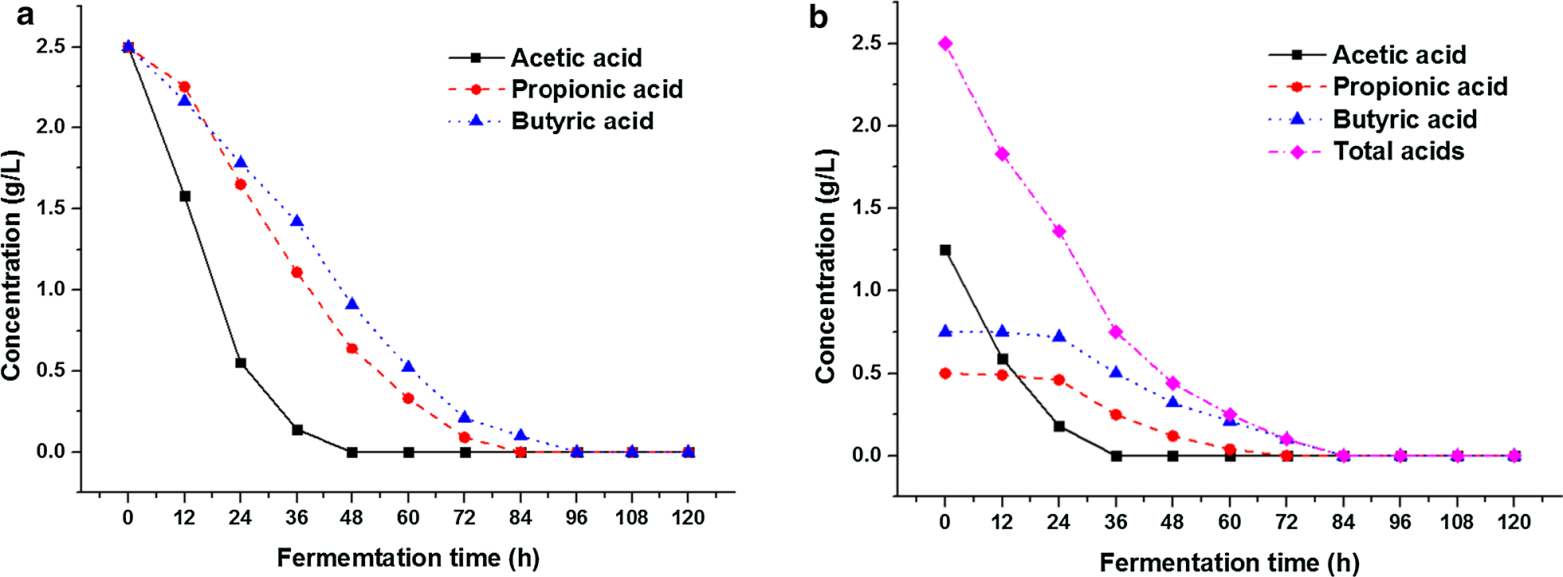
Time course of VFA utilization by *Y. lipolytica* on **a** 2.5 g/L single type VFA and **b** 2.5 g/L VFA mixture with ratio of 5:2:3

As shown in Fig. 3a, at the VFA concentration of 2.5 g/L, *Y. lipolytica* can adapt to each type of VFA as a carbon sources and use it for cell proliferation with almost no lag phase. However, utilization rates were different. *Y. lipolytica* showed a preference for acetic acid, and faster utilization rates of acetate over other VFA were confirmed in both single acid and mixed VFA systems. Slower utilization rates of propionate and butyrate to acetate were attributed to their different metabolic fates after intake. Unlike acetate, propionate and butyrate cannot be cleaved directly into acetyl-CoA, a central intermediate in lipid synthesis. As reported by Vajpeyi and Chandran [17], the rate of propionate metabolism is controlled by the rate of its decarboxylation to acetyl-CoA. In case of butyrate, the high number of biochemical transformations that it must undergo include β-oxidation to acetoacetyl-CoA, and then further cleavage to acetyl-CoA may be the cause of its slower uptake.

The utilization of volatile fatty acids by *Y. lipolytica* in a mixed VFA system is shown in Fig. 3b. As depicted, during the first 24 h, the utilization of propionic and butyric acid was in a state of stagnation. After exhaustion of acetic acid, the utilization of butyric acid by *Y. lipolytica* significantly accelerated. The results further illustrate an interesting finding, that is, in a mixed VFA system, the utilization of different types of VFAs by *Y. lipolytica* was not in a synchronized manner but rather in step-wise manner. *Y. lipolytica* would first use acetic acid for lipid production and then propionic and butyric acid. When sufficient acetate is available as carbon source, the metabolism of propionate and butyrate by *Y. lipolytica* will likely be suppressed or be halted.

### Cultivation of *Y. lipolytica* on VFA under the condition of high temperature

Comparative studies at 38 °C were carried out to evaluate the tolerance of *Y. lipolytica* for a high temperature environment with different types of VFA as carbon sources. Compared with propionic and butyric acids, the use of acetic acid as the sole carbon source improved the adaptability of *Y. lipolytica* to this higher temperature. At the same concentrations, cultures on acetic acid restored cell proliferation more rapidly. As shown in Table 2B, the lag phase of cultures on acetic acid under 38 °C was extremely close to those of corresponding cultures under 28 °C and were delayed by approximately 1 day. However, lag phase duration was markedly prolonged when propionic and butyric acids were used as carbon sources, especially at higher concentrations. Under the cultivation temperature of 38 °C, cultures failed to achieve effective cell growth and lipid production when more than 5 g/L propionic or butyric acid was used as the sole carbon source.

**Fig. 4:**
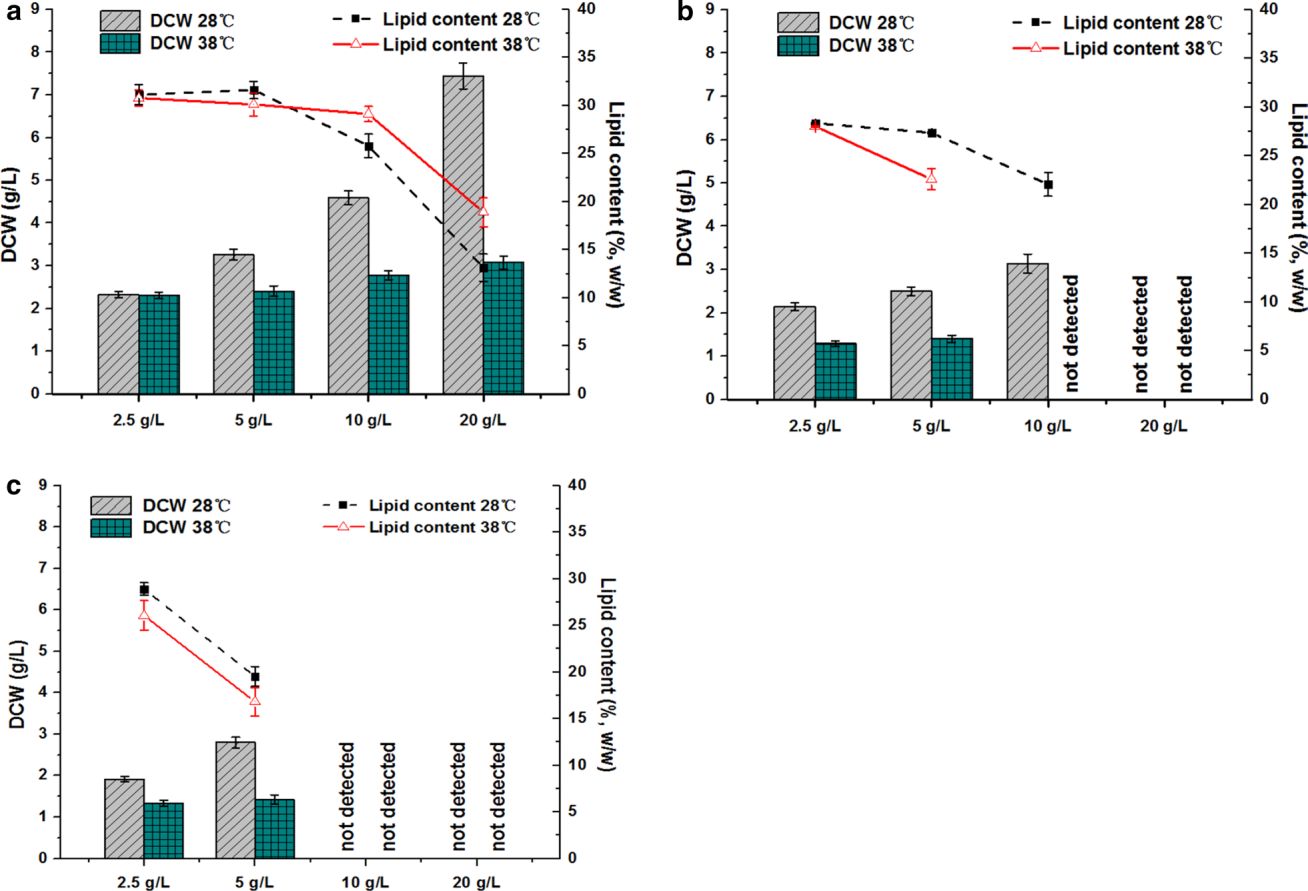
Comparison of dry cell weight (DCW) and lipid content obtained during batch cultivation with different concentrations of **a** acetic acid, **b** propionic acid, and **c** butyric acid under two different temperature conditions

Although *Y. lipolytica* could adapt to a high temperature of 38 °C and gradually restore cell proliferation, its measured DCW considerably decreased during biomass harvest (shown in Fig. 4). Moreover, cultures at higher VFA concentrations suffered higher losses in biomass production because of the inhibitory effect of a high volatile fatty acids concentration. For instance, the DCW of cultures on 5, 10, and 20 g/L acetic acid decreased by 26.16, 39.39, and 58.66%, respectively, compared with those of corresponding cultures at 28 °C. The lipid content of the cultures under 38 °C exhibited similar trends with those of cultures under 28 °C. Lipid production on acetic acid was superior to those on propionic and butyric acids. Moreover, relatively high final lipid contents were obtained at relatively lower VFA concentrations. However, unlike the drastic reduction in biomass, the decrease in lipid content value was relatively mild. The data in Fig. 4 indicated that for cultures on 10 and 20 g/L acetic acid, the measured lipid content even exceeded those of the corresponding cultures under 28 °C. This result was inconsistent with expectations. The effect of temperature on cell growth and lipid accumulation is a comprehensive result of various factors. To a certain extent, increasing temperature accelerates the metabolism of microbial cells. Thus, under high temperature conditions, yeast cells lost their cell integrity and catalytic efficiency in terms of cell multiplication, production of enzymes, and metabolic activities [23]. In this work, the dead and decomposed yeast cells released a portion of the residual lipid into the solution, as shown in Fig. 6c, thereby increasing the measured lipid content compared with the actual value.

### Cultivation of *Y. lipolytica* on VFA without pH adjustment

pH is a main factor that affects the cell growth and lipid accumulation of oleaginous microorganisms [8]. Different oleaginous microorganisms have different optimal pH culture conditions. Several researchers [1, 17] have reported that the lipid content of yeast cells is higher during the stationary phase when the initial pH value of the culture medium is close to neutral. Unlike traditional carbon sources, such as glucose and crude glycerol, the amount of volatile fatty acids used as carbon sources will directly affect system pH by increasing environmental acidity and decreasing the pH value. Environment acidity that exceeds a certain limit can damage microorganisms, destroy plasma membranes, inhibit enzyme activity, and disturb protein membrane transport. Therefore, pH regulation is an essential operation in the cultivation of oleaginous yeasts. Yeast cells have the ability to control the entry and exit of hydrogen ions into their cells to maintain a neutral intracellular environment. However, yeast cells also have some limits to their pH tolerance. Studying the tolerance of yeast strains for an acidic environment is important to obtain high concentration, high productivity, and high conversion yield in the application of volatile fatty acids as carbon sources. Strains with stronger acid tolerance can withstand a larger impact load of VFA concentration to ensure normal production. In addition, the wide tolerance of yeast to acidic pH could reduce the requirements of system pH regulation, thereby saving expenditures on alkali. Therefore, the comparative studies of batch cultures at different VFA concentrations without any pH adjustment were carried out to evaluate the tolerance and biomass and lipid production of *Y. lipolytica* in an acidic environment.

**Fig. 5:**
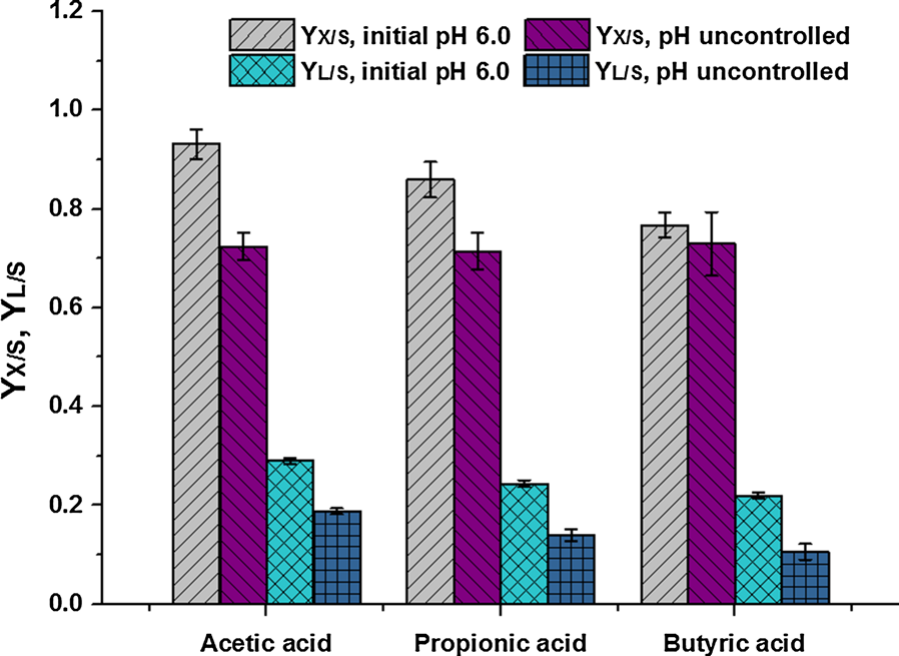
Comparison of growth yield coefficient (*Y*_*X*/*S*_) and lipid yield coefficient (*Y*_*L*/*S*_) obtained during batch cultivation on 2.5 g/L of VFAs under two different pH conditions

The initial pH of culture media without adjustment is shown in Table 3. All pH values were less than 4.0 at different VFA concentrations. *Y. lipolytica* has poor adaptability to this particular pH condition when using VFAs as carbon sources. During the 30-day observation period, only cultures on 2.5 g/L acetic, propionic, and butyric acids and 5 g/L acetic acid exhibited effective biomass and lipid production (Table 2C). Among these cultures, only the culture on 2.5 g/L acetic acid was able to quickly start cell growth within 24 h, however, its biomass and lipid production were obviously lower than cultures on 2.5 g/L glucose. The lag phases of *Y. lipolytica* cultures on 2.5 g/L propionic acid, 2.5 g/L butyric acid, and 5 g/L acetic acid were 6 ± 1, 17 ± 2, and 13 ± 2 days, respectively; these values were considerably longer than those of the corresponding cultures with an initial pH adjustment to 6.0. Moreover, the cell growth and lipid accumulation of *Y. lipolytica* was significantly inhibited. The *Y*_*X*/*S*_ and *Y*_*L*/*S*_ of cultures at an initial concentration of 2.5 g/L VFAs are shown in Fig. 5. Without an initial pH adjustment to 6.0, the *Y*_*X*/*S*_ of cultures on acetic, propionic, and butyric acids decreased from 0.931 ± 0.030, 0.859 ± 0.036, and 0.767 ± 0.026 by 22.2, 16.9, and 5.0% to 0.724 ± 0.028, 0.714 ± 0.037, and 0.729 ± 0.064, respectively. Meanwhile, *Y*_*L*/*S*_ decreased from 0.290 ± 0.006, 0.244 ± 0.007, and 0.219 ± 0.006 by 35.3, 42.6, and 51.6%, to 0.188 ± 0.006, 0.140 ± 0.012, and 0.106 ± 0.016, respectively. The microbial conversion of VFAs to lipid was severely inhibited. After a certain period of lag phase, *Y. lipolytica* could gradually adapt to these unfavorable pH conditions to survive and restore cell proliferation. However, the physiological activity of *Y. lipolytica* to convert VFAs into lipids was not ideal and likely weakened because certain enzymes that are involved in lipid synthesis were inhibited under this adverse environmental pH. Moreover, as the p*K*a of acetic, propionic, and butyric acid is 4.75, 4.87, and 4.82, respectively, these acids appear largely in undissociated form at acidic pH, which is more toxic than their dissociated form and exerted severe inhibitory effects [36].

**Table 3:** Initial pH of the culture medium with different VFA concentrations and without pH adjustment

VFA types	Acetic acid				Propionic acid				Butyric acid			
VFA Conc. (g/L)	2.5	5	10	20	2.5	5	10	20	2.5	5	10	20
pH value	3.80	3.64	3.42	2.51	3.92	3.77	3.58	2.63	3.96	3.81	3.69	2.68

As shown in Table 3, the pH values of culture media within the VFA concentration range of 2.5–20 g/L were not significantly different. By contrast, the lag phase, cell proliferation, biomass production, and lipid accumulation of the yeast were markedly different. However, these differences could not be simply attributed to the sensitivity of the cell growth and lipid accumulation of *Y. lipolytica* to changes in pH value. Complementary experiments (data not shown) indicated that cultures still had better performance at lower VFA concentrations despite adjusting pH values to the same low values with 2 mol/L HCl solution. Results further served as evidence and emphasized the inhibitory effect of high VFA concentrations on cell growth and lipid accumulation. Therefore, studying and establishing a suitable VFA concentration should receive more attention than fine and precise pH regulation in practical applications.

### Biomass and lipid production on food waste fermentate that contained VFA

The raw food waste used to prepare the fermenter feed had a total solids and total volatile solids content of (TS = 15.5 ± 1.1% w/w) and (VS = 14.2 ± 0.3% w/w), respectively. Elemental analysis of dried FW was carbon (41.7% DS), hydrogen (6.04% DS), oxygen (40.3% DS), nitrogen (3.02% DS), and sulfur (0.61% DS). The characteristics of the supernatant of FW fermentation effluent (before diluted) are shown in Table 4. The total VFA accounted 58.7 ± 7.3% of the SCOD. The most common VFAs present in the fermentate were acetate (45.3 ± 0.5%), propionate (18.1 ± 0.9%), and butyrate (29.2 ± 0.2%), all expressed as mass.

**Table 4:** Characteristics of the supernatant of Food waste fermentation effluent

Item	Concentration (g/L)
SCOD	49.29 ± 2.71
Total nitrogen	1.76 ± 0.18
Total VFA	28.94 ± 2.25
Acetic acid	13.10 ± 1.55
Propionic acid	5.24 ± 0.78
Butyric acid	8.43 ± 0.40
Valeric acid	0.79 ± 0.07
Isobutyric acid	0.41 ± 0.03
Isovaleric acid	0.97 ± 0.03

The concentration of the carbon and nitrogen sources in the effluent were measured after the solid content was removed by centrifugation and filtration. Three samples from the same experiment were analyzed

As shown in Table 5, the biomass concentration and the *Y*_*X*/*S*_ of cultures on synthetic VFA and the sterilized VFA stream from the anaerobic food waste fermenter were similar. However, the intracellular lipid content and the average lipid productivity were statistically lower for fermentate VFA. Cultures on synthetic VFA mixture obtained a lipid content of 31.69 ± 0.73% and an average lipid productivity of 0.23 ± 0.01 g/(L day) during the fermentation period, whereas the corresponding values on food waste fermentate were 18.23 ± 1.12% and 0.12 ± 0.02 g/(L day), respectively. Similarly, in previous studies by Vajpei et al. [17] and Chi et al. [37], the oleaginous yeast *C. albidus* and *C. curvatus* also showed a decrease in intracellular lipid content (14.9 and 13.5%) when using food waste fermentation effluent as the growth medium, thereby indicating the presence of as yet unknown inhibitors in the fermentate. Another possible cause of lower lipid yield could be the high protein content of the food waste, which can effectively increase the ammonium nitrogen concentration of the fermentate. The lipid accumulation process in oleaginous microorganisms could be divided into two types, namely, de novo lipid accumulation and ex novo lipid accumulation. Different from the lipid fermentation on hydrophobic substrates (ex novo lipid accumulation), de novo lipid accumulation (on the sugars or other hydrophilic substrates) usually requires limited nitrogen sources and relatively high C/N ratios [38]. In the present study, the C/N ratio of the FW fermentation effluent (C/N = 10.5) used for *Y. lipolytica* batch cultures was far below the favorable range (C/N:30–80) [39], which is unfavorable for de novo lipid accumulation.

**Table 5:** Biomass and lipid production of *Y. lipolytica* on food waste fermentate

Carbon source	Synthetic VFA mixture	VFA from anaerobic fermenter		
	(28 °C, 180 rpm, initial pH 6.0)	(28 °C, 180 rpm, initial pH 6.0)	(38 °C, 180 rpm, initial pH 6.0)	(28 °C, 180 rpm, pH uncontrolled)
DCW (g/L)	2.152 ± 0.102	2.029 ± 0.150	1.850 ± 0.177	1.667 ± 0.143
Lipid conc. (g/L)	0.682 ± 0.011	0.370 ± 0.027	0.334 ± 0.021	0.272 ± 0.035
Lipid content (wt %)	31.69 ± 0.73	18.23 ± 1.12	18.09 ± 0.89	16.33 ± 1.07
Y_X/S_	0.861 ± 0.041	0.812 ± 0.060	0.740 ± 0.071	0.667 ± 0.057
Y_L/S_	0.273 ± 0.004	0.148 ± 0.010	0.133 ± 0.008	0.108 ± 0.014
Lipid productivity g/(L days)	0.23 ± 0.01	0.12 ± 0.02	0.11 ± 0.02	0.07 ± 0.03

Furthermore, for the comparative cultures at 38 °C and cultures without pH control, both cultures began cell growth with extremely short lag phase (< 3 h), which is even better than cultures on synthetic VFA under the same culture conditions (round 1 day lag phase). On one hand, this result shows that the oleaginous yeast *Y. lipolytica* adapted well to this high temperature and low concentration acid environment when cultured in food waste fermentate. On the other hand, the result indicates that the sufficient nitrogen nutrition of food waste fermentate could have a promoting effect on the cell growth [38]. In addition, for cultures without pH adjustment, the high content of ammonia nitrogen improved the pH value of the fermentate (pH: 4.9–5.2), thereby making it more suitable for the growth of yeast.

**Fig. 6:**
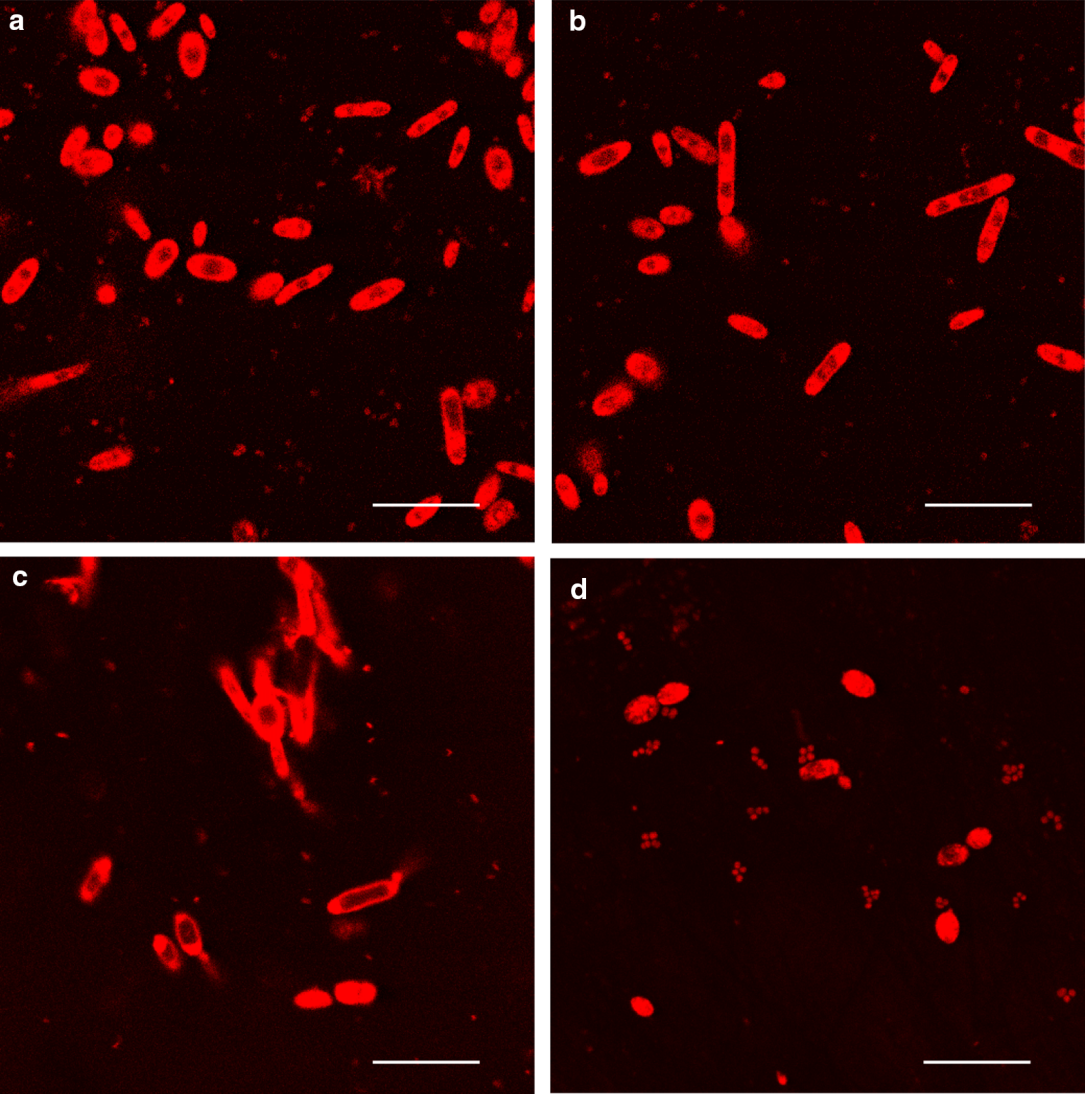
Confocal fluorescent images of *Y. lipolytica* cultured with 2.5 g/L of **a** synthetic VFA mixture at 28 °C, initial pH 6.0, **b** FW-derived VFA at 28 °C, initial pH 6.0, **c** FW-derived VFA at 38 °C, initial pH 6.0, **d** FW-derived VFA at 28 °C, pH uncontrolled. The scale bar is 18.75 μm

The above results indicate that the oleaginous yeast *Y. lipolytica* could utilize FW-derived VFA for lipid production and have a good tolerance for a high temperature or acidic environments; however, the lipid yield was lower than cultures on synthetic VFA. The results of fluorescent testing (Fig. 6) were also consistent with this conclusion. Future studies should be focused on the inhibitors in food waste fermentate and the metabolism of the yeast. In addition, a good process dealing with the specific C/N could have great potential to further scale up the lipid yield of *Y. lipolytica* on food waste fermentate.

### Analysis of fatty acid composition of lipids

The fatty acid composition of lipids obtained during batch cultivation of *Y. lipolytica* was determined by GC analysis after esterification (Table 6). In all cases with synthetic and food waste-derived VFA, the accumulated fatty acids of the lipids were predominantly palmitic (C16:0), stearic (C18:0), oleic (C18:1), and linoleic acids (C18:2) and correspond to those of vegetable and soybean oil [40], thereby suggesting that microbial lipid could be used as feedstock for biodiesel production. Similar fatty acid profiles of lipids that were synthesized by *C. albidus* and *C. curvatus* on a mixture of VFAs or acetic acid have been reported in the literature [1, 17, 41]. In addition, to achieve the best compromise between cold flow properties, that is, the tendency of fuel to solidify at lower temperatures, the optimum feedstock for biodiesel should have relatively low levels of saturated fats and high levels of unsaturated fats [42]. As demonstrated in Table 6, the fatty acids of the lipids obtained from *Y. lipolytica* on VFAs were mostly unsaturated, which are highly suitable for biodiesel production.

**Table 6:** Main fatty acid composition of the lipids produced by *Y. lipolytica* under different culture conditions

Culture condition	Carbon source	Relative fatty acid content (%)							
		C16:0	C16:1	C18:0	C18:1	C18:2	C18:3	Unsaturated	Total
28 °C, 180 rpm, initial pH 6.0	Acetic acid	13.3	3.9	10.5	43.6	21.6	2.3	75.0	95.2
	Propionic acid	15.2	7.1	12.7	35.2	20.6	1.9	69.9	92.7
	Butyric acid	14.9	5.2	16.6	33.5	19.9	1.2	65.5	91.3
	FW-derived VFA	20.5	4.3	18.7	34.1	7.6	0.8	46.8	86.0
38 °C, 180 rpm, initial pH 6.0	Acetic acid	21.7	5.5	19.0	24.6	14.2	N.D.	52.1	85.0
	Propionic acid	23.0	10.8	23.6	19.0	11.3	N.D.	46.9	87.7
	Butyric acid	20.5	9.3	25.2	15.9	11.6	N.D.	44.6	82.5
	FW-derived VFA	28.8	3.5	22.0	23.4	2.7	N.D.	29.6	80.4
28 °C, 180 rpm, pH uncontrolled	Acetic acid	17.9	5.6	13.0	37.5	17.9	0.6	66.6	92.5
	Propionic acid	20.0	9.7	16.4	30.2	14.1	N.D.	59.7	90.4
	Butyric acid	18.8	8.4	22.6	23.5	15.6	N.D.	53.4	88.9
	FW-derived VFA	18.9	4.2	18.3	30.9	5.5	N.D.	40.6	77.8

In cultures at higher temperatures (38 °C) or without pH adjustment, the content of unsaturated fatty acids in lipids decreased and that of saturated fatty acids increased. In particular, a higher temperature led to an obvious decline in the content of unsaturated fatty acids. Linolenic acid (C18:3) was undetected at the cultivation temperature of 38 °C. This result mainly occurs high temperature usually changes cellular membrane fluidity, and yeast adapts to this change by altering its fatty acid composition [43, 44].

In general, the content of unsaturated fatty acids of the lipids obtained from *Y. lipolytica* on food waste fermentate was lower than on synthetic VFA. In particular, the ratio of linoleic acid (18:2) would markedly decrease. The oleaginous yeast might use cellular lipid to maintain its growth when carbon source was used up or insufficiently supplied [38]. Hence, a possible reason of lower content of unsaturated lipids could be low C/N in the fermentate, which might lead the *Y. lipolytica* to selectively consume linoleic acid. In addition, the presence of as yet unknown inhibitors in the fermentate could also influence the yeast, and more metabolic level studies are needed.

## Conclusions

The study demonstrated the feasibility of using synthetic and food waste-derived VFA for lipid production by *Y. lipolytica*. The cell growth and lipid accumulation in *Y. lipolytica* using three typical types of VFA (acetic, propionic, butyric acid) as carbon sources were systematically compared and evaluated. For the first time, the inhibitory effect of each type of VFA was determined via prolonged culture duration. Moreover, the VFA utilization by *Y. lipolytica* in VFA mixture was elucidated as well. These results provided valuable information for using volatile fatty acids as substrates for lipid production.

Under optimal culture condition (28 °C, initial pH 6.0), the highest lipid content obtained with acetic, butyric, and propionic acids reached 31.62 ± 0.91, 28.36 ± 0.74, and 28.91 ± 0.66% of the dry mass at an initial concentration of 5, 2.5, and 2.5 g/L, respectively. High concentrations of VFA had inhibitory effect on cell growth in the following order: butyric acid > propionic acid > acetic acid. Within a 30-day experimental period, *Y. lipolytica* could adapt up to 20 g/L acetic acid, whereas the corresponding concentrations of propionic and butyric acid were 10 and 5 g/L. Cultures on a VFA mixture illustrated the utilization of different types of VFA by *Y. lipolytica* were not a synchronized but step-wise manner. *Y. lipolytica* would first use acetic acid for lipid production and then propionic and butyric acid. When sufficient acetate is available as carbon source, the metabolism of propionate and butyrate by *Y. lipolytica* will likely be suppressed or be halted. It is suggested that the acetate production dominant anaerobic process should be properly controlled to improve lipid yield downstream, especially in high load operation.

Given that yeast fermentation is an exothermic process and the addition of VFA will directly affect the pH of the system by increasing environmental acidity, the tolerance of the yeast for a high temperature and acidic environments was investigated. The results demonstrated that *Y. lipolytica* had good adaptabilities when using a low concentration (2.5 g/L) of either synthetic or food waste-derived VFA and the lipid composition was similar to commercial biodiesel feedstock, thereby further demonstrating the potential of *Y. lipolytica* to produce lipids from VFA. However, batch cultures fed with food fermentate, yielded a lower lipid content (ranged 16.3–18.5%) than cultures on synthetic VFA. Future studies should be focused on the inhibitors in food waste fermentate to make a better use of FW-derived VFA.

## Abbreviations

VFA: volatile fatty acids
DCW: dry cell weight
*Y*_*X*/*S*_: growth yield coefficient, g DCW/g VFAs
*Y*_*L*/*S*_: lipid yield coefficient, g lipid/g VFAs
rpm: rotations per minute
YPD: yeast peptone dextrose
FW: food waste
AD: anaerobic digestion
N.D.: not detected

## References

[1] Fei Q, Chang HN, Shang L, Choi JD, Kim N, Kang J (2011). The effect of volatile fatty acids as a sole carbon source on lipid accumulation by *Cryptococcus albidus* for biodiesel production. Biores Technol.

[2] Zhu LD, Hiltunen E, Antila E, Zhong JJ, Yuan ZH, Wang ZM (2014). Microalgal biofuels: flexible bioenergies for sustainable development. Renew Sustain Energy Rev.

[3] Ghaly AE, Dave D, Brooks MS, Budge S (2010). Production of biodiesel by enzymatic transesterification. Am J Bioresour Technol.

[4] Demirbas A (2009). Political, economic and environmental impacts of biofuels: a review. Appl Energy.

[5] Xu X, Kim JY, Cho HU, Park HR, Park JM (2015). Bioconversion of volatile fatty acids from macroalgae fermentation into microbial lipids by oleaginous yeast. Chem Eng J.

[6] Blatti JL, Michaud J, Burkart MD (2013). Engineering fatty acid biosynthesis in microalgae for sustainable biodiesel. Curr Opin Chem Biol.

[7] Meng X, Yang J, Xu X, Zhang L, Nie Q, Xian M (2009). Biodiesel production from oleaginous microorganisms. Renew Energy.

[8] Ratledge C (1991). Microorganisms for lipids. Acta Biotechnol.

[9] Ratledge C, Cohen Z (2008). Microbial and algal oils: Do they have a future for biodiesel or as commodity oils?. Lipid Technology.

[10] Vanessa B, Laurent P, Gwendoline C, Andre L, Christian L, Pierre F (2015). Improvement and modeling of culture parameters to enhance biomass and lipid production by the oleaginous yeast *Cryptococcus curvatus* grown on acetate. Biores Technol.

[11] Cescut J, Fillaudeau L, Molina-Jouve C, Uribellarea JL (2014). Carbon accumulation in *Rhodotorula glutinis* induced by nitrogen limitation. Biotechnol Biofuels.

[12] Yang X, Jin G, Gong Z, Shen H, Bai F, Zhao ZK (2014). Recycling biodiesel derived glycerol by the oleaginous yeast *Rhodosporidium toruloides* Y4 through the two-stage lipid production process. Biochem Eng J.

[13] Wu S, Zhao X, Shen H, Wang Q, Zhao ZK (2011). Microbial lipid production by *Rhodosporidium toruloides* under sulfate-limited conditions. Bioresour Technol.

[14] Pessiot J, Nouaille R, Jobard M, Singhania RR, Bournilhas A, Christophe G, Fontanille P, Peyret P, Fonty G, Larroche C (2012). Fed-batch anaerobic valorization of slaughterhouse by-products with mesophilic microbial consortia without methane production. Appl Biochem Biotechnol.

[15] Christophe G, Lara DJ, Kumar V, Nouaille R, Fontanille P, Larroche C (2012). Production of oils from acetic acid by the oleaginous yeast *Cryptococcus curvatus*. Appl Biochem Biotechnol.

[16] Fontanille P, Kumar V, Christophe G, Nouaille R, Larroche C (2012). Bioconversion of volatile fatty acids into lipids by the oleaginous yeast *Yarrowia lipolytica*. Bioresour Technol.

[17] Vajpeyi S, Chandran K (2015). Microbial conversion of synthetic and food waste-derived VFA to lipids. Biores Technol.

[18] Komemoto K, Lim YG, Nagao N (2009). Effect of temperature on VFA’s and biogas production in anaerobic solubilization of food waste. Waste Manage.

[19] Zhang YJ, Jiang JG, Wang JM (2013). Effect of pH value on VFA concentration and composition during anaerobic fermentation of kitchen waste. China Environ Sci.

[20] Han SK, Shin HS (2002). Enhanced acidogenic fermentation of food waste in a continuous-flow reactor. Waste Manage Res.

[21] Lim SJ, Kim BJ, Jeong CM (2008). Anaerobic organic acid production of food waste in once a day feeding and drawing-off bioreactor. Biores Technol.

[22] Banat IM, Nigam P, Singh D, Marchant R, Mchale AP (1998). Review: ethanol production at elevated temperatures and alcohol concentration: Part 1—Yeasts in general. World J Microbiol Biotechnol.

[23] Chen W (2008). To explore the influence of temperature on the yeast population. J Bull Biol.

[24] Magdalena R, Zbigniew L, Thierry D, Patrick F, Jean MN (2015). Lipid production by the oleaginous yeast *Yarrowia lipolytica* using industrial by-products under different culture conditions. Biotechnol Biofuels.

[25] Beopoulos A, Mrozova Z, Thevenieau F, Dall MT, Hapala I, Papanikolaou S (2008). Control of lipid accumulation in the yeast *Yarrowia lipolytica*. Appl Environ Microbiol.

[26] Papanikolaou S, Chevalot I, Komaitis M, Aggelis G, Marc I (2001). Kinetic profile of the cellular lipid composition in an oleaginous *Yarrowia lipolytica* capable of producing a cocoabutter substitute from industrial fats. Antonie Van Leeuwenhoek.

[27] Papanikolaou S, Chevalot I, Komaitis M, Marc I, Aggelis G (2002). Single cell oil production by *Yarrowia lipolytica* growing on an industrial derivative of animal fat in batch cultures. Appl Microbiol Biotechnol.

[28] Xie D, Jackson EN, Zhu Q (2015). Sustainable source of omega-3 eicosapentaenoic acid from metabolically engineered *Yarrowia lipolytica*: from fundamental research to commercial production. Appl Microbiol Biotechnol.

[29] Kolouchova I, Schreiberova O, Sigler K, Masak J, Rezanka T (2015). Biotransformation of volatile fatty acids by oleaginous and non-oleaginous yeast species. FEMS Yeast Res.

[30] APHA (2005). Standard methods for the examination of water and wastewater.

[31] Jiang J, Gong C, Wang J, Tian S, Zhang Y (2014). Effects of ultrasound pre-treatment on the amount of dissolved organic matter extracted from food waste. Bioresour Technol.

[32] Bligh E, Dyer WJ (1959). A rapid method of total lipid extraction and purification. Can J Biochem Physiol.

[33] Bourque SD, Titorenko VI. A quantitative assessment of the yeast lipidome using electrospray ionization mass spectrometry. J Vis Exp JoVE. 2009;30. doi:10.3791/1513 10.3791/1513 PMC3149914 19701157

[34] Morrison WR, Smith LM (1964). Preparation of fatty acid methyl esters and dimethylacetals from lipids with boron fluoride-methanol. Food Science and Technology.

[35] Rodrigues G, Pais C (2000). The influence of acetic and other weak carboxylic acids on growth and cellular death of the yeast *Yarrowia lipolytica*. Food Technol Biotechnol.

[36] Gong ZW, Shen HW, Zhou WT, Wang YD, Yang XB, Zhao ZK (2015). Efficient conversion of acetate into lipids by the oleaginous yeast *Cryptococcus curvatus*. Biotechnol Biofuels.

[37] Chi ZY, Zheng YB, Ma JW, Chen SL (2011). Oleaginous yeast *Cryptococcus curvatus* culture with dark fermentation hydrogen production effluent as feedstock for microbial lipid production. Int J Hydrogen Energy.

[38] Papanikolaou S, Aggelis G (2011). Lipids of oleaginous yeasts. Part II: technology and potential applications. Eur J Lipid Sci Technol.

[39] Moreton R, Moreton R (1988). Physiology of lipid accumulation yeasts. Sing cell oil.

[40] Zlatanov M, Pavlova K, Antova G, Angelova-Romova M, Georgieva K, Rousenova-Videva S (2014). Biomass production by antarctic yeast strains: an investigation on the lipid composition. Biotechnol Biotechnol Equip.

[41] Li XM, Xiong L, Chen XF, Huang C, Chen XD, Ma LL (2015). Effects of acetic acid on growth and lipid production by *Cryptococcus albidus*. Am Oil Chem Soc.

[42] Hoekman SK, Broch A, Robbins C, Ceniceros E, Natarajan M (2012). Review of biodiesel composition, properties, and specifications. Renew Sust Energy Rev.

[43] Suutari M, Liukkonen K, Laakso S (1990). Temperature adaption in yeast: the role of fatty acids. J Gen Microbiol.

[44] Lu LL, Tan JX, Zhang W, Ma W, Li YJ, Wang M, Su XD (2003). The adaptation of yeast’s cell membrane system to high temperature and high ethanol concentration. J Agric Univ Hebei.

[45] Xu X, Kim JY, Oh YR, Park JM (2014). Production of biodiesel from carbon sources of macroalgae, Laminaria japonica. Bioresour Technol.

[46] Zheng YB, Chi ZY, Ahring BK, Chen SL (2012). Oleaginous yeast for biofuel production: ammonia’s effect. Biomass Bioenergy.

[47] Fei Q, Chang HN, Shang L, Choi J (2011). Exploring low-cost carbon sources for microbial lipids production by fed-batch cultivation of *Cryptococcus albidus*. Biotechnol Bioprocess Eng.

